# Methods for the Thermal Stabilization of α-L-Rhamnosidase and Inactivation of β-Glucosidase in the Naringinase Complex from *Aspergillus niger*

**DOI:** 10.3390/molecules31132232

**Published:** 2026-06-25

**Authors:** Joanna Bodakowska-Boczniewicz, Zbigniew Garncarek

**Affiliations:** Department of Biotechnology and Food Analysis, Faculty of Production Engineering, Wroclaw University of Economics and Business, Komandorska 118/120, 53-345 Wrocław, Poland; zbigniew.garncarek@ue.wroc.pl

**Keywords:** erythritol, xylitol, sorbitol, α-L-rhamnosidase, β-glucosidase, hesperidin, hesperetin 7-O-glucoside

## Abstract

Naringinase is an enzyme complex composed of α-L-rhamnosidase and β-D-glucosidase, capable of deglycosylating flavonoids such as hesperidin. α-L-rhamnosidase converts hesperidin into rhamnose and hesperetin 7-O-glucoside (Hes-7-G), while β-D-glucosidase further hydrolyses Hes-7-G to hesperetin. Selective inactivation of β-D-glucosidase enables accumulation of Hes-7-G, a compound with higher water solubility and bioavailability than hesperidin or hesperetin, making it valuable for food and biotechnological applications. This study aimed to identify conditions allowing selective inhibition of β-D-glucosidase while preserving α-L-rhamnosidase activity for efficient Hes-7-G production. The effects of pH, temperature, and incubation time were investigated, together with the influence of polyols and sugars, including inositol, sucrose, glycerol, xylose, erythritol, xylitol, and sorbitol, on α-L-rhamnosidase thermostability. Among the tested additives, erythritol significantly improved α-L-rhamnosidase thermostability. The highest selectivity was achieved by incubating the enzyme in 1.4 M erythritol at 70 °C for 10 min, resulting in ~5% residual β-D-glucosidase activity and 50% α-L-rhamnosidase activity. Under these conditions, α-L-rhamnosidase activity exceeded β-D-glucosidase activity by more than 60-fold. Selective thermal inactivation of β-D-glucosidase in the presence of erythritol provides an effective strategy for producing Hes-7-G from hesperidin and may enhance flavonoid bioavailability for industrial applications.

## 1. Introduction

A diet rich in plant-based foods is a key component in preventing lifestyle-related diseases. Bioactive plant compounds, particularly flavonoids, possess powerful antioxidant properties that regulate redox homeostasis whilst also exhibiting anti-inflammatory, anticancer, and anti-ulcer effects, making them ideal food ingredients [1]. Despite the broad spectrum of therapeutic effects of flavonoids, their use is limited by poor absorption due to their low water solubility. Poor solubility results in low bioavailability of flavonoids and high variability in their absorption, which limits their use as food additives or dietary supplements [2,3]. Flavonoids are commonly found in plants in both aglycone and glycoside forms [4]. Glycosylation significantly affects the physicochemical properties and biological activity of flavonoids, although its impact depends on the type of attached sugar moiety [5]. In general, glucosylation and galactosylation enhance flavonoid water solubility, whereas rhamnosylation tends to reduce it. For instance, hesperetin 7-O-glucoside exhibits approximately 50-fold higher water solubility than hesperidin [6]. The type of sugar moiety attached to a flavonoid can influence the bioavailability of its aglycone form. In particular, flavonoid glucosides are generally absorbed more efficiently than rhamnosides or rhamnoglucosides [5]. Because flavonoid absorption is often limited, various strategies have been developed to improve their bioavailability, including enzymatic deglycosylation [5].

Hesperidin is a flavonoid commonly found in citrus fruits, primarily in oranges [7,8]. It is used in the food industry and can be obtained, amongst other sources, from orange peels in the citrus industry [7,9]. Hesperidin is a flavanone glycoside composed of the aglycone hesperetin attached to a rutinose moiety containing glucose and rhamnose linked by an α-1,6-glycosidic bond. Its enzymatic conversion to hesperetin can proceed through two different pathways. In one route, α-L-rhamnosidase cleaves the terminal rhamnose residue, generating hesperetin-7-O-glucoside (Hes-7-G), which is subsequently converted to hesperetin by β-D-glucosidase through the removal of glucose [4,9,10]. In the alternative route, β-D-glucosidase directly hydrolyzes hesperidin, producing hesperetin and rhamnose in a single step [4]. Hesperetin-7-O-glucoside has attracted considerable interest due to its potential as a precursor for the production of new sweeteners [5]. In addition, this compound displays greater biological activity than both hesperidin and hesperetin, including more potent inhibition of enzymes involved in carbohydrate digestion and cholesterol metabolism, which may contribute to antidiabetic and hypocholesterolemic effects [6,10]. Furthermore, Hes-7-G has been reported to exhibit stronger anti-*Helicobacter pylori* activity [10] and to possess vasodilatory and blood pressure-lowering [6,11]. Enzymatic bioconversion of hesperidin to Hes-7-G may alter its site of absorption and increase its bioavailability [10,12]. The bioavailability of rhamnose-containing flavonoids is entirely dependent on their hydrolysis by the gut microbiota [5]. The increased solubility of Hes-7-G compared to hesperidin and hesperetin is desirable for practical applications in the food or pharmaceutical industries, as it can improve bioavailability [10]. Flavonoid hydrolysis can be carried out chemically, which often leads to side reactions, or enzymatically, which is characterized by high selectivity, efficiency, and mild reaction conditions [4].

Naringinase, as a complex of enzymes comprising α-L-rhamnosidase and β-D-glucosidase, can be used for the deglycosylation of flavonoids [13,14]. To date, naringinase has been successfully used for the hydrolysis of hesperidin [4,6,10,15,16]. Deglycosylation of hesperidin exclusively by α-L-rhamnosidase, to compounds containing a glucoside residue, may increase their bioavailability. Therefore, inhibiting β-D-glucosidase activity in naringinase is important for improving the bioavailability of flavonoid glycosides. Since naringinase exhibits both catalytic activities, a partial purification process can be carried out to increase the selectivity of α-L-rhamnosidase [17]. The production of hesperetin glucoside requires specific inactivation of β-D-glucosidase whilst maintaining high α-L-rhamnosidase activity.

Increasing thermal stability is beneficial for most biotechnological applications of proteins. The activity of α-L-rhamnosidase, as a subunit of naringinase from *Aspergillus niger*, decreases significantly at temperatures above 55 °C, which severely limits its industrial application [18].

In recent years, the use of osmotically active molecules to increase the heat resistance of enzymes has gained importance due to the low cost, simplicity, and practical application of this method [6,19]. The most commonly used osmotically active substances include polyols, sugars, and amino acids. However, the choice of a suitable additive depends on the enzyme [20]. These naturally occurring osmolytes protect proteins from thermal inactivation by stabilizing thermally unfolded proteins [19].

To date, there are few reports in the literature concerning the stabilization of naringinase or α-L-rhamnosidase derived from *Aspergillus niger* using polyhydroxy compounds. It has been demonstrated that the presence of sorbitol significantly increases the thermal stability of α-L-rhamnosidase from *Aspergillus niger*, which translates into higher efficiency of hesperidin conversion [6]. Furthermore, separate studies have shown that the addition of sorbitol improves the thermal stability of α-L-rhamnosidase from *Aspergillus terreus*, which is used in the enzymatic conversion of rutin to isoquercitrin [19].

In studies aimed at improving the thermal stability of other enzymes, the following have also been used: erythritol [21,22,23,24], xylitol [23,24,25,26], sorbitol [6,23,24,26,27,28], mannitol [23,27], glycerol [24,27,28] and glycine [27].

Erythritol belongs to the family of sugar alcohols; it consists of only four carbon atoms and therefore has the lowest molecular weight of all sugar alcohols, which is associated with slightly different physical and chemical properties [29]. Certain microorganisms naturally produce it as an osmotic protective agent [30]. Erythritol is mainly used in the food, medical, and pharmaceutical industries, and its use as a food additive is growing rapidly [31]. Erythritol has a sweetness level of approximately 70% that of sucrose, but contains only 5% of the calories provided by sucrose [32]. It offers benefits such as being non-cariogenic and having no effect on blood sugar levels. In 1997, erythritol was recognized by the US Food and Drug Administration (FDA) as a food additive ‘generally recognized as safe’ (GRAS) [31].

This study aimed to determine optimal conditions for selective inhibition of β-D-glucosidase activity within the naringinase enzyme complex, whilst maintaining the maximum possible activity of α-L-rhamnosidase. This approach targeted the enzymatic deglycosylation of hesperidin to selectively obtain hesperetin 7-O-glucoside (Hes-7-G), a compound with superior solubility and bioavailability, with potential applications in the pharmaceutical and food industries. The key challenge is to select conditions for the selective inactivation of β-D-glucosidase whilst maintaining high activity of α-L-rhamnosidase in the naringinase preparation obtained from *Aspergillus niger* KMS.

To the best of our knowledge, no report has been published on the use of erythritol to improve the thermostability of α-L-rhamnosidase whilst simultaneously inactivating β-D-glucosidase, thereby enabling increased enzymatic conversion of hesperidin to Hes-7-G.

## 2. Results and Discussion

### 2.1. Effect of Polyhydroxy Compounds on the Stability of α-L-Rhamnosidase

The effect of selected polyhydroxy compounds on the thermal stability of α-L-rhamnosidase, a component of naringinase, was analyzed. Compounds frequently used as protein stabilizers include inositol, sucrose, glycerol, xylose, erythritol, xylitol, and sorbitol. The control sample consisted of naringinase incubated in McIlvaine’s buffer (pH 4.0) at 20 °C for 30 min. In the native enzyme, the ratio of α-L-rhamnosidase to β-D-glucosidase activity was 6.93.

The selection of an appropriate stabilizing agent depends on the enzyme’s structure. In this study, the addition of xylitol and sorbitol demonstrated significant (*p* < 0.05) protection against thermal denaturation compared with the control group, although varying degrees of stabilization were observed (Table 1).

After 30 min of incubating naringinase at 60 °C in the presence of 0.7 M xylitol and sorbitol, the activity of α-L-rhamnosidase remained significantly higher than that of the enzyme heated without the addition of a stabilizer (N). Similarly, the activity of α-L-rhamnosidase incubated in 0.7 M erythritol was higher than that of the enzyme without a stabilizer; however, the difference was not statistically significant. At the same time, the lowest β-D-glucosidase activity was observed in all three cases (Table 1). Furthermore, the addition of these polyols enabled a higher activity ratio between the subunits in the naringinase complex than in the other cases. An increase in the ratio of α-L-rhamnosidase to β-D-glucosidase activity was observed, from 6.93 to 7.01, 7.02, and 8.51, for erythritol, xylitol, and sorbitol, respectively.

These results indicate that the tested polyhydroxy compounds exhibit a protective effect on α-L-rhamnosidase activity. Consequently, erythritol, xylitol, and sorbitol were used in further studies. In the next stage of the research, the effects of selected polyhydroxy compound concentration, temperature, and incubation time on the inactivation of β-D-glucosidase and the simultaneous stabilization of α-L-rhamnosidase, as subunits of naringinase from *Aspergillus niger*, were investigated.

In accordance with the literature, the protective effect of polyhydroxy compounds is correlated with the increasing number of hydroxyl groups in the molecule [26], as confirmed by the experimental results. Sorbitol (6 –OH groups), xylitol (5 –OH groups), and erythritol (4 –OH groups) exhibit varying stabilizing efficacy, which is consistent with the observed protective effect on α-L-rhamnosidase.

Furthermore, other studies have shown that the presence of polyhydroxy compounds stabilizes glucoside dehydrogenase; at equal concentrations, the increase in the enzyme’s thermostability followed the order: glycerol < erythritol < xylitol < sorbitol [24].

Several studies in the literature indicate that polyhydroxy compounds enhance the thermal stability of enzymes. Ge et al. [19] demonstrated that the addition of sorbitol increases the thermostability of α-L-rhamnosidase from *Aspergillus terreus*, prolonging the enzyme’s half-life and increasing the activation energy of denaturation, thereby promoting both structural stability and catalytic efficiency at elevated temperatures (65–80 °C). Similarly, Sun et al. [6], found that the presence of sorbitol improves the thermostability of α-L-rhamnosidase from *Aspergillus niger* and increases the conversion of hesperidin to hesperetin-7-O-glucoside.

No publications were found in the literature regarding the effect of xylitol or erythritol on the thermostability of naringinase, α-L-rhamnosidase, or β-D-glucosidase. However, there is strong evidence suggesting that polyhydroxy compounds stabilize enzymes by protecting protein structures from thermal denaturation [23]. Studies on other proteins have shown that compounds such as xylitol, erythritol, and sorbitol can influence protein structure and stabilization mechanisms by modulating hydration and intramolecular interactions [22,25,26,33]. It has been demonstrated that erythritol alters the secondary and tertiary structures of trypsin. A reduction in β-sheet structure and an increase in α-helix were observed, reflecting the close relationship between the secondary structure elements and the enzyme’s biological activity. Molecular docking and thermodynamic studies confirmed the presence of hydrophobic interactions and hydrogen bonds between the erythritol molecule and the enzyme [22]. It may also apply to hydrolytic enzymes, such as α-L-rhamnosidase, subjected to high temperatures.

### 2.2. Effect of Temperature and Polyhydroxy Compounds on the Stability of α-L-Rhamnosidase

To determine the effect of inactivation temperatures in solutions of erythritol, xylitol, and sorbitol on the activity of naringinase subunits, the enzyme was heated for 10 min at elevated temperatures (60, 65, 70, 75, and 80 °C). All compounds studied showed a protective effect on α-L-rhamnosidase (Table 2). Activity was compared to that of enzymes incubated at the temperatures analyzed without the addition of polyhydroxy compounds (N).

Incubation of the enzymes for 10 min at 75 °C resulted in almost complete loss of α-L-rhamnosidase activity and complete denaturation of β-D-glucosidase. The best results (inactivation of β-D-glucosidase and simultaneous stabilization of α-L-rhamnosidase) were obtained when naringinase was incubated in a solution containing erythritol and xylitol at 70 °C (Table 2). The activity of α-L-rhamnosidase stabilized with erythritol and xylitol was 49.49% and 48.32%, respectively, of the initial activity of this enzyme. In both cases, the activity of β-D-glucosidase was negligible, accounting for approximately 6% of the initial activity. In incubation with sorbitol at 70 °C, the lowest α-L-rhamnosidase activity was observed among the tested compounds. In contrast, the highest β-D-glucosidase activity was observed at the same temperature.

At 70 °C, the highest activity ratio of both subunits was also observed (Table 3). For enzymes incubated in erythritol and xylitol, the activity ratio of α-L-rhamnosidase to β-D-glucosidase is 56.36 and 50.38, respectively. It is a significantly higher value than in native naringinase. The activity of β-D-glucosidase was strongly reduced, whilst the activity of α-L-rhamnosidase remained relatively high. The lowest activity of α-L-rhamnosidase and, at the same time, the highest activity of β-D-glucosidase stabilized with sorbitol resulted in the lowest ratio of the activities of both subunits among those studied, equal to 29.19. It is worth noting that a higher temperature alone alters the activities of the subunits and their activity ratio. There are no reports in the available literature on the selective thermal inactivation of one of the naringinase subunits whilst maintaining α-L-rhamnosidase activity, which prevents a direct comparison of the results.

Given the high activity ratio of both subunits, following the incubation of naringinase in a solution of erythritol and xylitol, these compounds were used in further studies. The effects of temperature and incubation time on the inactivation of β-D-glucosidase and the simultaneous stabilization of α-L-rhamnosidase, as subunits of naringinase from *Aspergillus niger*, were investigated.

Comparison of the results with literature data is limited. It has only been shown that the addition of 2.0 M sorbitol increases the thermostability of α-L-rhamnosidase from *Aspergillus terreus* in the temperature range of 60–85 °C [19]. At the same time, taking into account the half-life, the activation energy of the thermal inactivation process, and the potential practical applications of the enzyme, Ge et al. [19] studied *Aspergillus terreus* in the temperature range of 60–85 °C [19]. At the same time, taking into account the half-life, the activation energy of the thermal inactivation process, and the potential practical applications of the enzyme, Ge and colleagues [19] identified 70 °C as the optimal incubation temperature for α-L-rhamnosidase, which is consistent with the results of this study.

Furthermore, it has been shown that sorbitol prolongs the deactivation time and increases the thermostability of α-L-rhamnosidase from *Aspergillus niger* at 60 °C. Studies conducted on other enzymes, however, indicate that the protective effect of compounds such as sorbitol, glycerol, inositol, or glycine against laccase significantly decreases above 60 °C [21], i.e., at temperatures lower than those used in this study.

### 2.3. Effect of Erythritol and Xylitol Concentrations on the Stability of α-L-Rhamnosidase

**Figure 1:**
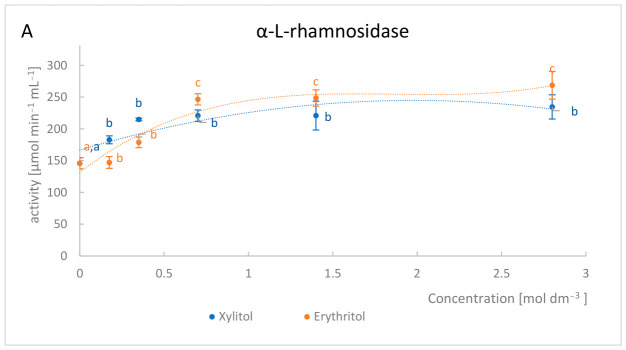

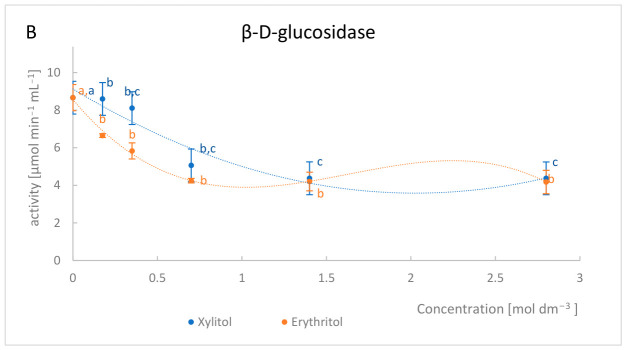
Effect of xylitol and erythritol concentrations on the activity of α-L-rhamnosidase (**A**) and β-D-glucosidase (**B**) as subunits of naringinase from *Aspergillus niger* KMS. Different lowercase letters within the same color indicate statistically significant differences between values (*p* < 0.05).

The effect of various concentrations of erythritol and xylitol on the activity of α-L-rhamnosidase and β-D-glucosidase at 70 °C was investigated (Figure 1A,B). As the concentration of erythritol or xylitol increased, the activity of α-L-rhamnosidase increased. The maximum retained activity of α-L-rhamnosidase, amounting to 52.46%, was achieved after the addition of 2.8 M erythritol. However, such a solution exhibits a high kinematic viscosity of 2.16 ± 0.03 mm^2^ s^−1^, which significantly impedes the hydrolysis reaction and the measurement of activity. Consequently, it was decided to use a final erythritol concentration of 1.4 M for further studies. α-L-rhamnosidase incubated for 10 min at 70 °C in a 1.4 M solution of erythritol or xylitol retained 48.52% and 42.12% of its initial activity, respectively.

It is difficult to compare these data with the literature. There are no studies in the literature on the effect of erythritol concentration on the activity of naringinase and its subunits. Ge et al. [19] studied the thermostability of α-L-rhamnosidase in the presence of sorbitol. It was shown that the thermostability of α-L-rhamnosidase increases with increasing erythritol concentration from 0.2 M to 2.5 M following incubation at 70 °C for 1 h. Sun et al. [6] investigated the effect of another polyhydroxy compound—sorbitol—on the activity of α-L-rhamnosidase. They demonstrated that after 30 min of incubation in a 1.2 M sorbitol solution, the relative activity of α-L-rhamnosidase was at its maximum. However, the maximum conversion of hesperidin to Hes-7-G, 52.7%, was achieved upon addition of 0.8 M sorbitol. As sorbitol concentration increased, hesperidin conversion gradually decreased. The researchers concluded that high sorbitol concentrations may reduce the mass transfer coefficient, which affects the contact between the enzyme and the substrate.

As the concentration of erythritol increases, the stability of trypsin also increases. The highest residual trypsin activity was observed upon incubation in a 2 M erythritol solution [22]. Different results were obtained with laccase. It was noted that a low concentration of the polyhydroxy compound has the greatest effect on improving the temperature stability of laccase in a highly hydrophilic polyol solution [21]. Sorbitol at 0.6 M provided the best protection for laccase over the temperature range 20–60 °C.

### 2.4. Effect of Incubation Time in Xylitol and Erythritol Solutions on the Stability of α-L-Rhamnosidase

**Figure 2:**
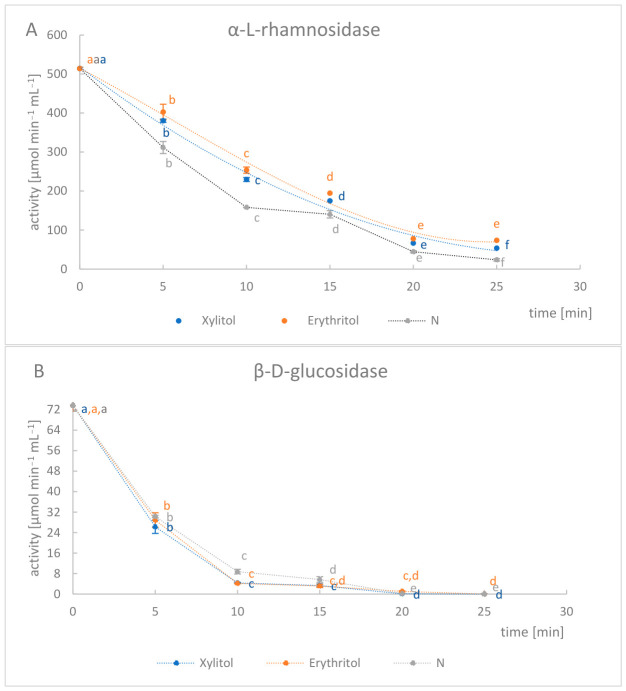
Effect of the incubation time of *Aspergillus niger* KMS naringinase in xylitol and erythritol solutions on the activity of α-L-rhamnosidase (**A**) and β-D-glucosidase (**B**). N—enzyme heated at 70 °C for a specified time, without the addition of a stabilizer. Different lowercase letters within the same color indicate statistically significant differences between values (*p* < 0.05).

The effect of the incubation time of naringinase from *Aspergillus niger* KMS on the activity of α-L-rhamnosidase and β-D-glucosidase was investigated at 70 °C in 1.4 M solutions of erythritol or xylitol (Figure 2A,B). Analysis of the data presented indicates that α-L-rhamnosidase activity decreased systematically with increasing incubation time, regardless of the polyhydroxy compound used. At the same time, both xylitol and erythritol exhibited a protective effect compared to the control sample, with erythritol showing slightly higher enzyme activity over the analyzed time range. It was particularly significant that after 10 min of incubation, relatively high α-L-rhamnosidase activity and trace β-D-glucosidase activity were still observed. As a result, almost complete inactivation of β-D-glucosidase was achieved (residual activity ~4%), and nearly 50% of α-L-rhamnosidase activity was retained. Under these conditions, α-L-rhamnosidase was over 60 times more active than β-D-glucosidase, which enabled the targeted hydrolysis of hesperidin to Hes-7-G (Table 4). Upon extending the naringinase incubation time to 20 min in 1.4 M solutions of xylitol and erythritol, a significant decrease in enzymatic activity was observed. After 20 min of naringinase incubation, the activity of α-L-rhamnosidase decreased to 12.96% in the xylitol solution and 15.15% in the erythritol solution, respectively, whilst the activity of β-D-glucosidase was close to zero. It resulted in very high activity ratios for the two subunits. However, due to the significant reduction in α-L-rhamnosidase activity, it was concluded that the optimal incubation time for naringinase in xylitol and erythritol solutions is 10 min.

Sun et al. [6] demonstrated that the conversion of hesperidin reached its maximum level due to α-L-rhamnosidase following the addition of 0.70 M sorbitol at 60 °C and pH 4.5 after 10 min. Incubation of xylanase with a 2 M solution of xylitol and sorbitol at 52 °C demonstrated a protective effect of both polyhydric alcohols for 300 min, compared to the control sample [26]. It is a significantly longer duration, but also a much lower temperature than in the case of the presented research results.

Due to the high ratio of α-L-rhamnosidase to β-D-glucosidase activity in the naringinase complex and the negligible activity of β-D-glucosidase, incubation with an erythritol solution (1.4%) was selected as a method to inactivate β-D-glucosidase whilst maintaining relatively high α-L-rhamnosidase, for targeted hydrolysis leading to the production of Hes-7-G, incubation with a solution of erythritol (1.4 M) at 70 °C for 10 min was selected. 

### 2.5. Hydrolysis of Hesperidin Contained in Model and Fresh Orange Juice

**Figure 3:**
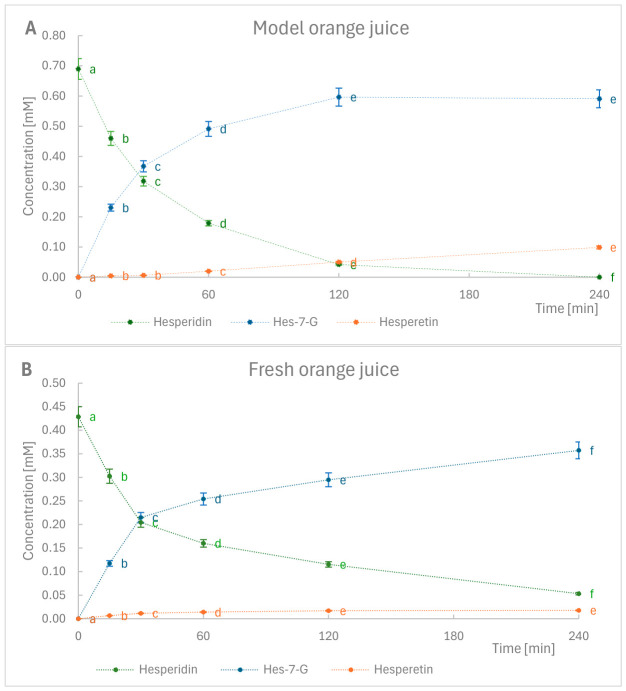
Hydrolysis of hesperidin in model juice (**A**) and fresh orange juice (**B**) by modified naringinase from *Aspergillus niger* KMS. The reactions were carried out at 20 °C. Different lowercase letters within the same color indicate statistically significant differences between values (*p* < 0.05).

Naringinase, modified by incubation with erythritol (1.4 M) at 70 °C for 10 min, was used to hydrolyze hesperidin present in fresh and model orange juice. Hesperidin hydrolysis was carried out at 20 °C. The contents of hesperidin, Hes-7-G, and hesperetin in the model juice and fresh orange juice were determined by high-performance liquid chromatography (Supplementary Materials (Figure S1)). The content of hesperidin, Hes-7-G, and hesperetin in the juice was determined before hydrolysis, as well as after 15, 30, 60, 120, and 240 min of reaction time. In both systems studied, the model solution and fresh orange juice, effective conversion of hesperidin to Hes-7-G was observed, confirming the high activity of α-L-rhamnosidase (Figure 3A,B). In the model juice, the reaction proceeds more rapidly; after just 2 h of continuous hydrolysis, almost 94% of the hesperidin contained in the model solution had been hydrolyzed. The conversion of hesperidin to Hes-7-G by α-L-rhamnosidase was over 86%; only about 7% of Hes-7-G underwent further hydrolysis by β-D-glucosidase.

The initial hesperidin content in the hydrolyzed orange juice was 0.43 mM. The results show that enzymatic bioconversion of hesperidin by a modified naringinase in fresh orange juice leads to a gradual increase in Hes-7-G content. After 2 h of hydrolysis, depending on the hesperidin concentration used, 69% Hes-7-G and just under 4% hesperetin were obtained. In freshly squeezed orange juice, the process proceeds more slowly than in the model juice; after 4 h of hydrolysis, approximately 12% of hesperidin remains in the juice. At the same time, the amount of hesperetin formed is significantly lower (approx. 4%), which may suggest additional inhibition of β-D-glucosidase activity by natural components of orange juice.

The efficiency of hesperidin hydrolysis also depends on the stability and activity of enzymes in the target environment, which is acidic fruit juice. The low pH of orange juice (pH = 3.2), which is lower than the optimal pH value determined for naringinase and its subunits from *A. niger* KMS (pH = 4.0 [18]), may affect the hydrolytic activity of the enzyme in orange juice. In addition to pH, several factors, such as different substrates, osmolarity, and potential endogenous inhibitors, may reduce enzyme activity in fresh orange juice [34].

There are a few studies in the literature concerning the hydrolysis of hesperidin contained in fresh orange juice. Researchers often focus on the hydrolysis of hesperidin in a model system, often conducting the reaction at the optimal temperature for the enzyme in question.

Sun et al. [6] increased the thermostability of α-L-rhamnosidase from *Aspergillus niger* by adding 0.7 M sorbitol. At pH 4.5 and 60 °C, with a hesperidin concentration of 0.5 mM, they demonstrated that after 30 min, the conversion of hesperidin to Hes-7-G was 63.26%.

Lee et al. [10] carried out the hydrolysis of hesperidin using naringinase from *A. sojae*, characterized by high α-L-rhamnosidase activity and relatively low β-D-glucosidase activity, to convert it into the intermediate product Hes-7-G. The reaction was conducted at 37 °C. The hesperidin in orange juice and peel was effectively converted by the α-L-rhamnosidase from naringinase into a glucoside, with a small amount of the aglycone hesperetin being produced. The yield of Hes-7-G, relative to the amount of hesperidin present in the extracts, was estimated at approximately 71% for orange juice and 78% for orange peel, which is slightly lower than the yield described in the present study.

Hesperidinase from *Penicillium* sp., possessing both α-L-rhamnosidase and β-D-glucosidase activity, was used to hydrolyze the hesperidin contained in orange juice (181.2 μg/mg of hesperidin). After 4 h of reaction, 60% of the hesperidin contained in the juice had been hydrolyzed to hesperetin. The reaction was carried out at 40 °C [34].

## 3. Materials and Methods

### 3.1. Materials

The study used a naringinase preparation obtained from deep-bed cultivation of *Aspergillus niger* KMS, as described in the previous article [35]. After separating the mycelial biomass from the culture medium, a liquid enzyme preparation was obtained with a naringinase activity of 1.05 ± 0.08 µmol·min^−1^·mL^−1^. The α-L-rhamnosidase activity in the obtained naringinase complex was 520.58 ± 13.28 µmol·min^−1^·mL^−1^, and the β-D-glucosidase activity in the obtained naringinase complex was 72.80 ± 3.80 µmol·min^−1^·mL^−1^.

p-nitrophenyl-α-L-rhamnopyranoside (pNPR) and p-nitrophenyl-β-D-glucopyranoside (pNPG) were purchased from Sigma-Aldrich (St. Louis, MO, USA). Polyhydroxy compounds: inositol (Austranal Praparate, Fischamend, Austria), sucrose (Chempur, Piekary Śląskie, Poland), glycerine (POCH, Gliwice, Poland), xylose (Merck, Darmstadt, Germany), erythritol (Biomus, Lublin, Poland), xylitol (Krüger, Ostrów Mazowiecki, Poland), sorbitol (Biomus, Lublin, Poland).

Studies on the hydrolysis of hesperidin were conducted using model juice and fresh orange juice. The model juice consisted of 400 µg·mL^−1^ of hesperidin, 0.48% sucrose, and 0.025% citric acid. Fresh orange juice was obtained from Navel Powell oranges (country of origin: Spain) purchased at a local supermarket. Freshly squeezed orange juice contained 338 µg· mL^−1^ of hesperidin.

### 3.2. Analytical Methods

#### 3.2.1. Determination of α-L-Rhamnosidase and β-D-Glucosidase Activity from Naringinase

The activities of α-L-rhamnosidase and β-D-glucosidase were determined according to the method of Spagna et al. [36], based on spectrophotometric measurement of p-nitrophenol released from p-nitrophenyl-α-rhamnopyranoside (pNPR) or p-nitrophenyl-α-glucopyranoside (pNPG) at 410 nm. The reaction mixture contained 0.4 mL^3^ of 2 mM substrate solution in 0.1 M acetate buffer (pH 4.0), 0.01 mL of appropriately diluted enzyme solution, and 0.09 cm^3^ of water. After incubation for 5–10 min, the reaction was terminated by adding 1 mL^3^ of 1 M Na_2_CO_3_. Enzyme activity was expressed as µmol of pNPR or pNPG hydrolyzed per minute by 1 mL of enzyme solution.

#### 3.2.2. Effect of Polyhydroxy Compounds on the Stability of α-L-Rhamnosidase and β-D-Glucosidase

The effect of selected polyhydroxy compounds (inositol, sucrose, glycerol, xylose, erythritol, xylitol, sorbitol) on the thermal stability of α-L-rhamnosidase was assessed. Solutions of the test compounds were prepared in McIlvaine’s buffer (pH 4.0).

1 mL of naringinase was incubated with 0.5 mL of the test compound solution for 30 min at 60 °C. The final concentration of the tested polyhydroxy compounds in the reaction mixture was 0.7 M. Additionally, under the same conditions, 1 mL of naringinase and 0.5 mL of McIlvaine’s buffer (pH 4.0) were incubated without the addition of stabilizers (control N). The control sample consisted of naringinase incubated in McIlvaine’s buffer (pH 4.0) at 20 °C for 30 min, without the addition of the test compounds (sample 0). After incubation, the samples were immediately cooled in an ice bath to approximately 5 °C. The activity of α-L-rhamnosidase and β-D-glucosidase was then determined.

#### 3.2.3. Effect of Naringinase Incubation Temperature in Solutions of Polyhydroxy Compounds on the Stability of α-L-Rhamnosidase and β-D-Glucosidase

1 mL of naringinase was incubated for 10 min with 0.5 mL of a solution of erythritol, xylitol, and sorbitol, prepared in McIlvaine’s buffer (pH 4.0), at temperatures of 60, 65, 70, 75, and 80 °C. The final concentration of the tested polyhydroxy compounds in the reaction mixture was 0.7 M.

The control sample consisted of naringinase incubated in McIlvaine’s buffer (pH 4.0) at 20 °C for 10 min, without the addition of the tested compounds (sample 0). Additionally, for each tested temperature, the enzyme activity incubated for 10 min in McIlvaine’s buffer (pH 4.0) without stabilizing agents was determined (sample N). After incubation, the samples were immediately cooled in an ice bath. The activity of α-L-rhamnosidase and β-D-glucosidase was then determined.

#### 3.2.4. Effect of Erythritol and Xylitol Concentrations on the Stability of α-L-Rhamnosidase and β-D-Glucosidase

1 mL of naringinase was incubated for 10 min at 70 °C with 0.5 mL of a solution of erythritol and xylitol, prepared in McIlvaine’s buffer (pH 4.0). Various concentrations of the compounds in the reaction mixture were tested: 0.175 M, 0.35 M, 0.7 M, 1.4 M, and 2.8 M.

The control sample consisted of naringinase incubated in McIlvaine’s buffer (pH 4.0) at 20 °C for 10 min, without the addition of the compounds under investigation (sample 0). Additionally, the activity of the enzyme incubated for 10 min at 70 °C in McIlvaine’s buffer (pH 4.0), without the addition of stabilizing agents, was determined (sample N). After incubation, the samples were immediately cooled in an ice bath. The activity of α-L-rhamnosidase and β-D-glucosidase was then determined.

#### 3.2.5. Effect of Incubation Time in Xylitol and Erythritol Solutions on the Stability of α-L-Rhamnosidase and β-D-Glucosidase

1 mL of naringinase was incubated for 10 min at 70 °C in 0.5 mL of erythritol and xylitol solutions, prepared in McIlvaine’s buffer (pH 4.0). The activity of α-L-rhamnosidase and β-D-glucosidase was determined after 5, 10, 15, 20, and 25 min of incubation of naringinase in solutions of the tested polyhydroxy compounds. The final concentration of the tested polyhydroxy compounds in the reaction mixture was 1.4 M.

The control sample consisted of naringinase incubated in McIlvaine’s buffer (pH 4.0) at 20 °C for 10 min, without the addition of the tested compounds (sample 0). Additionally, the enzyme activity incubated at 70 °C in McIlvaine’s buffer (pH 4.0) without stabilizing agents for a specified time was determined (sample N). After incubation, the samples were immediately cooled in an ice bath. The activity of α-L-rhamnosidase and β-D-glucosidase was then determined.

#### 3.2.6. Hydrolysis of Hesperidin Contained in Orange Juice

The hydrolysis of hesperidin was carried out in model and fresh orange juice using naringinase, which had been modified by incubation with erythritol (1.4 M) at 70 °C for 10 min. A total of 13 mL of freshly squeezed orange juice and 2 mL of enzyme (treated with 1.4 M erythritol) were mixed in a conical flask and incubated at 20 °C. The concentrations of hesperidin were determined in the juice before hydrolysis and at 15, 30, 60, 120, and 240 min after the start of the reaction. After treatment with the free enzyme, the reaction was stopped by heating the mixture to boiling for 5 min. All samples were filtered through Whatman filters. The hesperidin concentration in grapefruit juice was analyzed by high-performance liquid chromatography (HPLC). Separation was performed on a C-18 analytical column (Chromolith™ Performance RP-18e) at 288 nm at a flow rate of 1 mL min^−1^ at room temperature. The injection volume was 20 µL. The mobile phase consisted of A: 3% acetic acid in water, B: 3% acetic acid in water: acetonitrile (1:1). Elution was carried out in a gradient with the following composition: 0–15 min: 25–33% B, 15–25 min: 60% B, 25–30 min: 25% B. All determinations were performed in triplicate. The LP-Chrom ver.1.54 software was used for the analysis of the chromatograms

#### 3.2.7. Measurement of the Viscosity of Polyhydroxy Compound Solutions

Viscosity measurements were carried out using a Ubbelohde viscometer (K = 0.01025 mm^2^ s^−2^) at 25 °C. For each sample, three independent replicates were performed, in which the time taken for the liquid to flow through the viscometer capillary was measured.

### 3.3. Statistical Analysis

Experiments were performed in triplicate, and results are presented as means ± standard deviation. Significance of differences (*p* < 0.05) was analyzed by analysis of variance (ANOVA) and Duncan’s test using STATISTICA v14.3 (TIBCO Software Inc., Palo Alto, CA, USA).

## 4. Conclusions

Increasing enzyme stability is one of the most difficult challenges due to several factors. Efforts to increase the stability of naringinase and its subunits are highly beneficial for reducing the costs of their industrial use.

From a practical standpoint, thermal stability is one of the most important characteristics to consider when using enzymes in industrial processes. In the case of naringinase and its application for the hydrolysis of hesperidin and the production of the intermediate product—Hes-7-G—an additional challenge is maintaining high activity in only one of its subunits, α-L-rhamnosidase, while simultaneously determining the optimal conditions for the selective inhibition of β-D-glucosidase activity. The thermal stability of the α-L-rhamnosidase from *Aspergillus niger* KMS naringinase was improved by adding polyhydroxy compounds, such as erythritol. It was demonstrated that the addition of erythritol improved the thermal stability of the α-L-rhamnosidase, whilst not protecting the β-D-glucosidase. The addition of erythritol can effectively improve the catalytic activity of α-L-rhamnosidase from naringinase, thereby making the enzyme more suitable for the biotransformation of hesperidin. It allowed for the effective conversion of hesperidin to Hes-7-G, yielding only a negligible amount of hesperetin. It was established that incubating naringinase with erythritol (1.4 M) at 70 °C for 10 min is a stable method, allowing for the targeted hydrolysis of hesperidin to Hes-7-G. It is confirmed by the high ratio of α-L-rhamnosidase to β-D-glucosidase activity in the naringinase complex from *Aspergillus niger* KMS and the negligible β-D-glucosidase activity. These results are promising for industrial applications of this versatile biological catalyst.

In the next stage of the research, it is worth characterizing in detail the naringinase complex and its subunits incubated in a solution with erythritol under optimal conditions. It will be important to determine the half-lives of naringinase and its subunits as a function of temperature, activation energy, and the activation energy of the thermal deactivation process of the modified enzymes. There is also a need to extend research on erythritol-induced stabilization of α-L-rhamnosidase, accounting for the enzyme’s conformational stability and structure using spectroscopic methods or molecular docking.

## Figures and Tables

**Table 1:** Effect of selected polyhydroxy compounds on the activity of α-L-rhamnosidase and β-D-glucosidase as subunits of naringinase from *Aspergillus niger* KMS.

	α-L-Rhamnosidase		β-D-Glucosidase		α-L-Rhamnosidase:β-D-Glucosidase Activity Ratio
	Activity [µmol min^−1^ mL^−1^]	Relative Activity [%]	Activity [µmol min^−1^ mL^−1^]	Relative Activity [%]	
0	518.36 ± 16.56 ^a^	100.00	74.80 ± 2.05 ^a^	100.00	6.93
N	348.61 ± 22.77 ^b^	67.25	56.60 ± 9.04 ^b^	75.67	6.16
Inositol	343.82 ± 11.88 ^b^	66.33	59.35 ± 5.60 ^b,c^	79.67	5.79
Sucrose	338.05 ± 5.79 ^b^	65.22	69.33 ± 0.10 ^a,c^	92.68	4.88
Glycerin	318.00 ± 11.55 ^b^	61.35	59.84 ± 13.65 ^b,c,d^	79.99	5.31
Xylose	342.11 ± 14.42 ^b^	66.00	60.68 ± 1.86 ^b,c,d^	81.11	5.64
Erythritol	357.33 ± 2.82 ^b,c^	68.94	51.00 ± 1.76 ^b,d^	68.18	7.01
Xylitol	386.10 ± 38.28 ^c^	74.49	55.02 ± 6.97 ^b,d^	73.56	7.02
Sorbitol	436.24 ± 14.15 ^d^	84.16	51.26 ± 1.38 ^b,d^	68.53	8.51

0—unheated enzyme, without stabilizer; N—enzyme heated at 60 °C for 30 min, without stabilizer. Different lowercase letters within the same column indicate statistically significant differences between values (*p* < 0.05).

**Table 2:** Effect of incubation temperature in solutions of polyhydroxy compounds on the activity of α-L-rhamnosidase and β-D-glucosidase as subunits of naringinase.

**α-L-Rhamnosidase**								
	**N**		**Xylitol**		**Erythritol**		**Sorbitol**	
	**Activity** **[µmol min^−1^ mL^−1^]**	**Relative Activity [%]**	**Activity** **[µmol min^−1^ mL^−1^]**	**Relative Activity [%]**	**Activity** **[µmol min^−1^ mL^−1^]**	**Relative Activity [%]**	**Activity** **[µmol min^−1^ mL^−1^]**	**Relative Activity [%]**
60 °C	453.62 ± 26.73 ^a^	85.90	469.45 ± 22.11 ^a^	88.90	464.05 ± 28.98 ^a^	87.88	524.32 ± 21.09 ^b^	99.29
65 °C	397.75 ± 13.94 ^a^	75.32	444.21 ± 27.34 ^b^	84.12	452.50 ± 10.49 ^b^	85.69	435.80 ± 8.81 ^b^	82.53
70 °C	156.53 ± 3.54 ^a^	29.64	255.15 ± 8.50 ^b^	48.32	261.36 ± 10.21 ^b^	49.49	232.77 ± 5.15 ^c^	44.08
75 °C	4.16 ± 0.64 ^a^	0.79	6.21 ± 0.44 ^a^	1.18	4.32 ± 0.06 ^a^	0.82	6.37 ± 4.46 ^a^	1.21
80 °C	0.00 ± 0.00 ^a^	0.00	0.00 ± 0.00 ^a^	0.00	0.00 ± 0.00 ^a^	0.00	0.00 ± 0.00 ^a^	0.00
**β-D-glucosidase**								
	**N**		**Xylitol**		**Erythritol**		**Sorbitol**	
	**Activity** **[µmol min^−1^ mL^−1^]**	**Relative Activity [%]**	**Activity** **[µmol min^−1^ mL^−1^]**	**Relative Activity [%]**	**Activity** **[µmol min^−1^ mL^−1^]**	**Relative Activity [%]**	**Activity** **[µmol min^−1^ mL^−1^]**	**Relative Activity [%]**
60 °C	72.68 ± 8.66 ^a^	96.24	75.17 ± 7.55 ^a^	99.54	73.88 ± 3.20 ^a^	97.83	71.96 ± 9.92 ^a^	95.30
65 °C	48.12 ± 7.03 ^a^	63.72	57.61 ± 6.61 ^a^	76.29	56.42 ± 5.61 ^a^	74.71	51.11 ± 7.04 ^a^	67.68
70 °C	11.61 ± 1.05 ^a^	15.37	5.06 ± 1.21 ^b^	6.71	4.97 ± 0.48 ^b^	6.14	7.97 ± 0.50 ^c^	10.56
75 °C	0.00 ± 0.00 ^a^	0.00	0.00 ± 0.00 ^a^	0.00	0.00 ± 0.00 ^a^	0.00	0.00 ± 0.00 ^a^	0.00
80 °C	0.00 ± 0.00 ^a^	0.00	0.00 ± 0.00 ^a^	0.00	0.00 ± 0.00 ^a^	0.00	0.00 ± 0.00 ^a^	0.00

N—enzyme heated at the test temperature for 10 min, without the addition of a stabilizer. In the unheated sample, without the addition of polyhydroxy compounds, the activity of α-L-rhamnosidase was 528.06 ± 6.17 µmol min^−1^ mL^−1^, and β-D-glucosidase was 75.20 ± 1.38 µmol min^−1^ mL^−1^, which corresponds to 100% enzyme activity. Different lowercase letters within the same row indicate statistically significant differences between values (*p* < 0.05).

**Table 3:** Ratio of α-L-rhamnosidase to β-D-glucosidase activity as subunits of naringinase incubated in solutions of polyhydroxy compounds as a function of temperature.

	α-L-Rhamnosidase:β-D-Glucosidase Activity Ratio		
	60 °C	65 °C	70 °C
N	6.24	8.27	13.49
Xylitol	6.24	7.71	50.38
Erythritol	6.28	8.02	56.36
Sorbitol	7.29	8.53	29.19

N—enzyme heated at the test temperature for 10 min, without the addition of a stabilizer. In the unheated sample, without the addition of polyhydroxy compounds, the ratio of α-L-rhamnosidase to β-D-glucosidase activity was 6.99.

**Table 4:** Ratio of α-L-rhamnosidase to β-D-glucosidase activity in solutions of xylitol or erythritol, depending on incubation time.

	α-L-Rhamnosidase:β-D-Glucosidase Activity Ratio			
	5 min	10 min	15 min	20 min
N	10.29	18.00	24.66	233.64
Erythritol	14.48	52.84	52.41	557.53
Xylitol	13.93	60.65	61.29	72.96

N—enzyme heated at 70 °C for a specified time, without the addition of a stabilizer. In the unheated sample, without the addition of polyhydroxy compounds, the ratio of α-L-rhamnosidase to β-D-glucosidase activity was 6.98.

## Data Availability

The data presented in this study are available at https://doi.org/10.18150/0HSLGJ.

## Supplementary Materials

The following supporting information can be downloaded at: https://www.mdpi.com/article/10.3390/molecules31132232/s1, Figure S1: Representative HPLC chromatogram showing the hydrolysis of hesperidin present in model juice.

## Abbreviations

The following abbreviations are used in this manuscript:

Hes-7-G	hesperetin 7-O-glucoside
pNPR	p-nitrophenyl α-L-rhamnopyranoside
pNPG	p-nitrophenyl-β-D-glucopyranoside
