# Supplementary material for: Methods for the Thermal Stabilization of α-L-Rhamnosidase and Inactivation of β-Glucosidase in the Naringinase Complex from Aspergillus niger
Source: Molecules. 2026 Jun 25;31(13):2232. doi: 10.3390/molecules31132232 (PMC13363437; doi:10.3390/molecules31132232)
Supplement: Supplementary file 1 [file molecules-31-02232-s001.zip › molecules-4345713-supplementary.pdf]

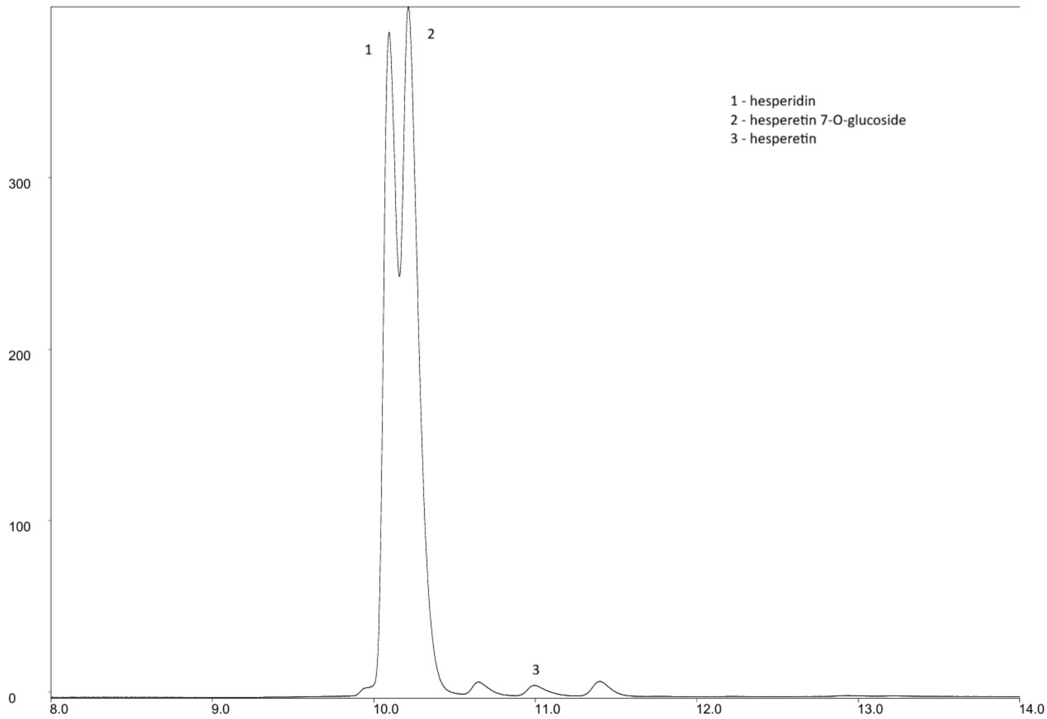

Figure S1: Representative HPLC chromatogram showing the hydrolysis of hesperidin present in model juice.
